# Cultivating the Ethical Repertoires of Behavior Analysts: Prevention of Common Violations

**DOI:** 10.1007/s40617-020-00540-w

**Published:** 2021-03-23

**Authors:** Lisa N. Britton, Amy A. Crye, Linda K. Haymes

**Affiliations:** 1 Britton Behavioral Consulting, PO Box 956, Pinole, CA 94564 USA; 2 Behavior Services of the Rockies, Lafayette, CO USA; 3 Touro University California, Graduate School of Education, Vallejo, CA 94592 USA

**Keywords:** Ethics, Behavior analysis, The BACB Code

## Abstract

Violations of the *Professional and Ethical Compliance Code for Behavior Analysts* occur despite coursework, supervision, and training. In this discussion, we highlight the most common violation categories identified: (a) improper or inadequate supervision/delegation, (b) failure to report/respond to the Behavior Analyst Certification Board (BACB) as required, and (c) professionalism/integrity. The specific areas addressed under supervision/delegation involve behavior analysts’ standards and performance as supervisors, as well as compliance with coursework. For failure to report, the focus is on responding, reporting, and providing updated information to the BACB in a timely manner. Finally, the section on professionalism and integrity addresses multiple code elements, including integrity, professionalism, and scientific relationships, as well as methods for promoting an ethical culture and decisions involving ethical violations by others. Importantly, we provide guidance on the structure and organization of supervision, methods and guidelines regarding reporting, and rubrics to shape and evaluate professionalism and integrity. We provide recommendations for the supervision process and for practitioners from the organizational perspective so that the organization supports and promotes an ethical culture.

In 2010, the Behavior Analyst Certification Board (BACB) brought ethics subject matter experts together to review current standards, and through that process, the BACB *Professional and Ethical Compliance Code for Behavior Analysts* (hereafter referred to as the BACB Code) was developed (BACB, 2014; Bailey & Burch, 2016). The BACB Code, which came into effect on January 1, 2016, gave greater clarity regarding the ethical practices of behavior analysts. The updates reflected the changes that had occurred within the behavior-analytic community, including an increased focus on the ethical behavior of the supervisor, as well as behavior analysts’ obligation to the BACB itself.

In the summer of 2018, the BACB released a white paper that outlined an analysis of the ethics violations during 2016 and 2017. Across those 2 years, the BACB accepted and resolved 219 notices and took action on 94 of them (BACB, 2018a). The most common violation categories identified within this report include (a) improper or inadequate supervision/delegation, (b) failure to report/respond to the BACB as required, and (c) professionalism/integrity. Upon further reflection on the specific BACB Code violations identified within this document, one can find that the issues around supervision and delegation reflected violations of BACB Code Element 5.0, related to behavior analysts as supervisors, as well as Code Element 10.05, which focuses on compliance with BACB supervision and coursework standards. Concerns related to failure to report/respond to the BACB highlighted challenges with Code Element 10.02, which addresses timely responding, reporting, and updating information provided to the BACB. Finally, concerns related to professionalism and integrity covered several code elements, including (a) 1.04 on integrity, (b) 1.05 related to professional and scientific relationships, (c) 7.01 regarding promoting an ethical culture, and (d) 7.02 regarding ethical violations by others and risk of harm (BACB, 2018a).

As a profession, behavior analysts may wonder whether 94 ethics violations across a 2-year span is concerning. One way to determine this is to compare these data with other professions that are similar to ours. We attempted to gain these statistics from the American Speech-Language-Hearing Association and the American Psychological Association (APA), but they do not release these data publicly. However, a review of the California Board of Psychology *Newsletter* (2016a, b, c, 2017) provides information regarding violations by individual psychologists. For California alone, in that 1 year, a total of 43 licensees required action due to serious violations. For comparison, there were 16,900 psychologists in California in 2018 (APA, 2020), whereas there were 4,645 Board Certified Behavior Analysts (BCBAs) in California in that same time period (BACB, 2020a). The actions ranged from probation, revocation from failing probation, and surrender. No analysis was provided on areas of violation; however, only the more serious violations that resulted in actions were presented. The offenses included, but were not limited to, gross negligence, dual relationships, inaccurate billing, drug convictions, driving under the influence, failure to get consent, and protecting confidentiality.

Regardless of the comparison across professions, most behavior analysts would agree that identifying ways to decrease violations is an important goal. Therefore, the purpose of this discussion is to identify ways to assist BCBAs in maintaining ethical behavior as it relates to the four areas of (a) improper or inadequate supervision/delegation, (b) how organizations can support effective supervision, (c) failure to report/respond to the BACB as required, and (d) professionalism/integrity as identified within the white paper published by the BACB in 2018 (BACB, 2018a). These areas are examined from the perspective of teaching and reinforcing ethical behavior, developing systems that support ethical behavior, and maintaining an ethical culture to ensure that these behaviors maintain over time.

## Improper or Inadequate Supervision/Delegation

Supervision within the field of behavior analysis is much more challenging than it appears if one is to do it well and within the bounds of the BACB Code. There are several components that make this complex. Specifically, one should ensure that they are competent in the area of focus for the trainee, that they are competent as a supervisor, that they consistently use evidence-based strategies within their supervision practice, and that they maintain the organizational skills necessary to meet the expectations set by the BACB related to the structure and paperwork surrounding supervision. In addition, the process for delegating tasks to trainees needs to occur with a careful balance to ensure that one is teaching the skills that trainees will need as BCBAs without giving them tasks to complete when they do not have the requisite skills to complete those tasks.

### Deciding to Provide Supervision

Britton and Cicoria (2019) discussed the daunting challenge that a BCBA goes through as one decides whether or not to start supervising trainees. Although there are many benefits to providing supervision—including increasing the skills of the supervisor, compensation, and building the skills of the next generation of behavior analysts—there are also challenges to contemplate. Specifically, a potential supervisor will want to consider the time commitment necessary to provide quality supervision. There is much more to supervision than scheduling weekly meetings and observations. Significant planning needs to occur for the supervision meeting to be effective. One concern is related to Code Element 5.02, which discusses supervisory volume (BACB, 2014). A BCBA should consider if they have the time to commit to quality supervision. Bailey and Burch (2016) highlighted that supervision volume should be at a level at which the trainee is able to adequately improve their skills as a result of the supervision provided.

Given that supervisors have an obligation to trainees, trainees’ clients, and the field as a whole, another consideration is to determine if one’s knowledge of the task list and the specific area of concentration for that trainee is sufficient to impart those skills to others. Ethical supervisors consider Code Element 1.01, which states that behavior analysts only provide services within their areas of competence (BACB, 2014). But how do BCBAs know if they are providing services—and in this case, supervision—within their areas of competence? Scope of competence consists of those activities that a specific practitioner has the skills to perform (Brodhead, Cox, & Quigley, 2018a). One recommendation stated that BCBAs consider their education, training, and supervised experience to determine their level of competence related to the task list (Brodhead, Cox, & Quigley, 2018a). The same would apply to the specific area of focus for potential trainees based on their place of employment. Brodhead, Cox, and Quigley (Brodhead, Cox, & Quigley, 2018a) also recommended that BCBAs evaluate the degree to which their clients have improved as a result of their efforts within that specific area of expertise within the field. Finally, it is helpful for BCBAs to assess whether they have kept up with advances in the field related to that area through their ongoing professional development when evaluating competence (Brodhead, Cox, & Quigley, 2018a). For example, if a trainee is working for a school district conducting functional behavior assessments, the supervisor will need to assess whether they have the skills to support the trainee within this context based on the aforementioned criteria.

Having competence in specific aspects of the task list is a start, but it is insufficient when determining if one is prepared to supervise trainees who are taking the steps necessary to sit for the exam. A BCBA should also assess the degree to which they have the prerequisite skills to supervise competently, as highlighted in Code Element 5.01 (BACB, 2014). Supervision competence includes the ability to use behavior skills training (BST) effectively to build the knowledge and application of behavioral principles with clients. Parsons, Rollyson, and Reid (2012) outlined a six-step process to BST: (a) describing the skill, (b) providing a written description of the skill, (c) modeling the skill, (d) providing an opportunity for rehearsal, (e) delivering performance feedback, and (f) continuing the process until a previously identified level of competency is achieved. This also aligns with Code Element 5.04, which emphasizes the importance of designing effective supervision that incorporates evidence-based training strategies that are behavior analytic in nature (BACB, 2014).

Given that 67 of the actionable violations identified in the white paper on the topic of ethics violations from 2016 and 2017 are related to inadequate supervision, it is clear that supervisors should consider these key areas when determining whether they are prepared to take on the significant responsibilities associated with supervision (BACB, 2018a). The areas discussed in this section are highlighted in Table 1, which identifies specific actions of a supervisor to support the provision of supervision in an ethical manner. The areas of focus as they relate to this section include the caseload volume of the supervisor, competence, and designing effective training that is behavior analytic in nature.

**Table 1:** Supervision decision process

Considerations	BACB code element	Actions
Time/volume • Current caseload evaluation	5.02 Supervisory Volume	• Set policy in the organization regarding number of cases.
• Current supervisory load		• Develop a caseload policy on percentage of time supervised vs. caseload.
• Other roles and commitments		• Develop a supervision schedule. • Develop job description for supervision.
• Personal commitments and circumstances	1.05(f) Professional and Scientific Relationships	• Conduct a self-assessment regarding personal commitments. • Utilize an organization professional dispositions rubric.
Competence • Education	5.01 Supervisory Competence	• Assess specialty areas of study or concentration.
• Training and background	1.02 Boundaries of Competence	• Create a list of proficiencies and fluency from the self-assessment to determine growth areas.
• Continuing education	1.03 Maintaining Competence Through Professional Development	• Provide access within the organization or paid time for continuing education.
• Conferences	1.03 Maintaining Competence Through Professional Development	• Select sessions and workshops that meet current developments in the area of competency.
• Access to journals/research	1.01 Reliance on Scientific Knowledge	• Read research on evidence-based procedures.
• Specialty or boutique areas		• Select workshops that expand current areas of expertise.
Baseline skill of task list • Technical skills	5.04 Designing Effective Supervision and Training	• Evaluate coursework.
		• Develop a scope and sequence of training curriculum. • Develop a job model of needed skills.
• Baseline analysis of ethics		• Assess official transcript. • Evaluate ethics coursework compared to current ethics requirements. • Assess verbal self-report regarding knowledge of ethics. • Utilize behavior skills training (BST) to build ethics repertoire.
Trainee	1.06 Multiple Relationships and Conflicts of Interest	• Assess relationship with trainee, coworker, employee, student, and community member for potential conflicts of interest.
Contract	10.05 Compliance With BACB Supervision and Coursework Standards	• Outline scope and sequence.
• Communication to determine terms of supervision	5.05 Communication of Supervision Conditions	• Develop a contract prior to start. • Check for agreement and understanding.
• Telehealth	2.06 Maintaining Confidentiality	• Ensure technology is compliant with the Health Insurance Portability and Accountability Act (HIPAA). • Assess video and technology skills. • Ensure family and insurance approval documentation is completed.
Delegation	5.03 Supervisory Delegation	• Perform BST to the level of independence.
		• Review key artifacts such as data, functional behavior assessment, and behavior intervention plan.
		• Create a timeline for development of program protocols.
		• Create a protocol for delegating duties to staff.
Feedback	5.06 Providing Feedback to Supervisees	• Link corrective feedback to action plans.
		• Ensure remote viewing occurs and meets HIPAA requirements.
		• Utilize BST during feedback sessions.
Differences	1.05(c) Professional and Scientific Relationships	• Seek training, consultation, conversations, and mentors.
Evaluation	5.07 Evaluating the Effects of Supervision	• Take social validity measures from stakeholders.
		• Review direct measures and graphs of client progress.
		• Evaluate checklists and rubrics regarding trainee performance.

### Using Evidence-Based Strategies in Supervision

Once a BCBA makes the decision to supervise, the next step is to develop systems to design supervision and training that include behavior-analytic content as outlined in Code Element 5.04 (BACB, 2014). The supervisor should be using behavioral procedures when designing their curriculum (Bailey & Burch, 2016). We recommend developing a scope and sequence that aligns with the task list to ensure that the supervisor is covering important concepts that a trainee needs to be effective. Although it is important for supervisors to expose trainees to all areas of the task list, it is unlikely that a trainee is going to reach a level of mastery on every aspect of the task list. Therefore, supervisors should evaluate which task list areas are of greatest priority for mastery. One recommendation is to use a job model to identify the skills that a trainee will need to perform within a specific organization (Garza, McGee, Schenk, & Wiskirchen, 2018). In the event that the supervisor does not work within the organization and does not have access to this information, it is still possible to identify the critical components that will be necessary by interviewing the trainee regarding the roles and responsibilities of BCBAs within the organization.

Once the supervisor has identified the areas of focus within supervision, the next step is to assess the trainee on these areas. Garza et al. (2018) highlighted the importance of assessing trainees’ current performance in these areas for two reasons. First, this will provide a baseline for the supervisor to be able to assess the effects of supervision, and second, it will allow for an efficient process for training, as the supervisor can focus training specifically on the deficits identified through the assessment process. The supervisor could carry out the assessment by using a treatment integrity checklist or rubric that identifies the key components of a particular skill, observing the trainee engaging in that skill, and rating the trainee on whether they engaged in each of the components identified. Research has demonstrated the effectiveness of using treatment integrity checklists in combination with performance feedback to increase the fidelity of treatment delivery (Witt, Noell, LaFleur, & Mortenson, 1997). Britton and Cicoria (2019) provided examples of rubrics that can be useful for this purpose. Given that Code Element 5.07 highlights the importance of evaluating the effects of supervision, this baseline becomes a relevant component of the supervision process (BACB, 2014).

Once the assessment is complete, the supervisor can begin training on the areas identified. These trainings typically include the BST steps identified previously within this article, as outlined by Parsons et al. (2012). The treatment integrity checklists or rubrics used during the initial assessment can be used throughout the training process. By aligning trainings to meet the standards of BST, the supervisor is ensuring that they are meeting the expectations outlined in Code Element 5.04, related to designing effective supervision and training, and Code Element 5.06, related to providing feedback to trainees (BACB, 2014). Table 1 further describes strategies that can be used to design effective supervision and training and evaluate the effects of supervision, as well as methods for providing feedback to trainees.

### Structure and Organization in Supervision

Organizational skills are an important component for any supervisor. The 2018 white paper identified Code Element 10.05, which focuses on compliance with BACB supervision and coursework standards, as an area of concern (BACB, 2018a). Examples of these standards are (a) developing a contract, (b) documenting the supervision process correctly, and (c) meeting experience requirements including the number of contacts and observations, the percentage of time that needs to be supervised, acceptable activities, and the percentage of time engaged in unrestricted activities (BACB, 2019a).

#### Supervision Contract

Developing and reviewing the supervision contract is the first step in starting a supervision relationship. The BACB Code highlights the importance of communicating the supervision expectations within Code Element 5.05 (BACB, 2014). The BACB website contains sample contracts that can be adjusted to meet the specific needs of the supervision context. Garza et al. (2018) made specific recommendations about reviewing the contract with a trainee, including reading the contract out loud, pausing at the end of each section to answer questions, and initialing each section once reviewed to document that both parties fully understand the content within that section. Table 1 provides additional detail regarding the supervision contract and methods for ensuring clear communication regarding the content of that contract.

As the supervisor and trainee continue the supervision relationship, it will be important to ensure that both parties uphold the expectations set forth in the contract. Sellers, LeBlanc, and Valentino (Sellers, LeBlanc, & Valentino, 2016a) provided recommendations for detecting and addressing concerns in the supervision relationship. These authors highlighted the importance of addressing issues as soon as they appear. They discussed the risk of the supervisor engaging in avoidance behaviors, such as ignoring the problems or discontinuing the supervision relationship, as opposed to addressing the concerns directly with the trainee (Sellers, LeBlanc, & Valentino, 2016a).

#### Documenting Supervision

The BACB has specific requirements regarding how the supervisor documents supervision, including the use of a unique documentation system, the completion of monthly verification forms, and the completion of the final verification form, to name a few (BACB, 2019a). Given that these requirements change over time, we will not cover the specifics of documenting. Given that these requirements change, it is important for supervisors to stay abreast of these changes, as well as when the changes go into effect. Bailey and Burch (2016) stated that the responsibility of ensuring that all supervision activities meet the expectations set forth by the BACB rests on the supervisor, and this includes documentation.

Developing systems and expectations around documentation is an important component to assist with organizational skills. We recommend including information regarding the documentation process within the supervision contract and utilizing the same process across trainees to ensure that nothing gets missed.

#### Experience Requirements

The experience requirements identify what the fieldwork experience should look like. Examples of this include the duration of the experience, the number of hours associated with each experience type, acceptable activities, and the percentage of hours engaged in unrestricted activities (BACB, 2019a). It is important for supervisors to have a full understanding of the BACB’s expectations around these requirements. As with documentation, these requirements change over time, and it is the supervisor’s responsibility to be aware of these changes and when they go into effect.

What can be especially challenging is when a supervisor is working with a trainee who has the potential to meet the requirements leading up to the point at which those requirements change. The BACB has provided guidance to ensure that trainees meet the requirements during the transition period leading up to the changes coming into effect on January 1, 2022 (BACB, 2020b). It will be important for supervisors to ensure that a trainee’s hours will count regardless of when they apply to sit for the exam.

### Delegation During the Supervision Process

Delegating tasks to a trainee should occur with care. It is important to ensure that the trainee has the requisite skills to perform that skill with competence. By using a BST method of training, supervisors can identify specific points at which they can fade their models and allow for a greater level of independence. Even when a trainee demonstrates independence with a skill, continued performance monitoring should occur to ensure generalization across clients and settings (Garza et al., 2018).

## Promoting Adequate Supervision and Delegation at the Organizational Level

As identified in the white paper, inadequate supervision within organizations is a frequently occurring ethical violation. During collaboration, we shared our experiences. Each of us agreed that across our careers, we commonly encountered concerns with adequate supervision. Hartley, Rosswourm, and LaMarca (2016) identified inconsistent learning opportunities for trainees as an area of concern for an organization. The organization has responsibilities to the trainee and to other stakeholders to provide appropriate supervision with systems in place to ensure consistency of supervision across supervisors and trainees. By entering into a contract of supervision with a trainee, a representative of the organization agrees to uphold appropriate levels of supervision and support across supervisors within the organization. Hartley et al. (2016, p. 330) noted that organizations would benefit from creating a “successful and mutually beneficial supervision model.” As such, it may be important for organizations to work to establish protocols to determine who should provide supervision and how that supervision should occur.

### Technical Competencies

Although self-assessment of competence is a needed ability, the organization can provide support in assessing the skills of employed behavior analysts and the development of those skills. Passage of the BCBA exam demonstrates a minimal level of competence. It does not guarantee that a BCBA is competent to supervise. Beginning in January 2022, new BCBAs will be required to either be in practice for 1 year before providing supervision or receive monthly consultation during this 1st year from a qualified consulting supervisor (BACB, 2017c). This is the minimum requirement to provide supervision. Turner, Fischer, and Luiselli (2016) noted that supervision requires the acquisition, refinement, and maintenance of skills. To address this, we suggest that the organization provide a mentor during the 1st year, and for as long as necessary, to build the skills the new BCBA needs to be a successful supervisor and to document the acquisition of these technical skills using rubrics and treatment integrity checklists.

The organization can further assist in the endeavor of assessment of skills by establishing periodic competency checks to ensure that supervisors can demonstrate the skills in the areas in which they are tasked to supervise. For best results, these competency checks can be provided on an ongoing basis. Competency checks should be performed at regularly scheduled intervals by an individual in the agency who is a BCBA, has demonstrated exceptional performance in the areas being assessed, has demonstrated superior supervision skills, and has scored highly on the disposition rubric discussed later in this article. Each organization should determine how often these checks should be performed depending on the needs and skill levels of supervisors, as well as the organization’s resources. An organization may decide to initially implement checks frequently (e.g., monthly) and then move to a leaner schedule (e.g., quarterly) for individuals who receive high scores. By completing these checks, the organization can ensure the supervisor has maintained their skills and has continued to be in contact with new research and developments. In the event that an assigned supervisor does not have adequate expertise, these areas of supervision would be reassigned to a supervisor with a command of the specified area, whether that supervisor is someone within the organization or outside the organization. It is suggested that organizations monitor the BACB Registry to ensure supervisors maintain supervisory status and that they monitor the continuing education (CE) of supervisors.

**Fig. 1:**
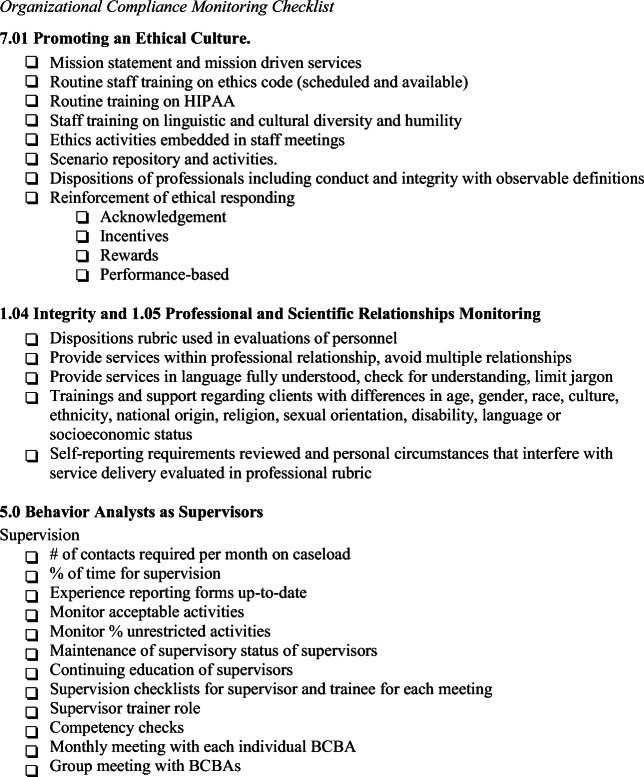
Organizational compliance monitoring checklist

Although it is the responsibility of the supervisor to ensure their ongoing qualifications, the organization may avoid pitfalls by assisting in this process. Figure 1, an organizational compliance monitoring checklist, identifies important considerations for monitoring the ethical practices of the organization, including the delivery of supervision. It is suggested that the leadership team of the organization use this checklist before initiating services (planning for the organization) and as part of their overall development plan for the organization. Some items on the checklist are essential for the onset of business. Examples of these include establishing a mission statement, mission-driven services, and a plan for initial and ongoing staff training, including training on ethics. Other components may be part of the development plan for the organization. The organization may review the checklist to identify areas of need that can be used for strategic planning and the development of organizational goals. Once these are established, the organization should continue to monitor practices on a monthly or quarterly basis to ensure progress on identified goals.

### Disposition

Even when a behavior analyst has extensive technical expertise in an area, there is no assurance that they will be a good supervisor. Before being considered to be a supervisor, the BCBA should demonstrate that they uphold the values of the organization, as well as the ethical standards of the BACB. More important than ever is the need to recognize and respect cultural diversity. The BACB acknowledges this, as do many members of the behavior-analytic community. A recent petition to the BACB called for “training behavior analysts on how to effectively work with individuals from diverse backgrounds” (Beaulieu, 2020). Although this broad area of need has not yet been explicitly identified in the 5th edition of the BACB Task List (BACB, 2017a), it is identified in Code Element 1.05(c) (BACB, 2014).

Table 2, the professional dispositions rubric, outlines additional areas that may be evaluated. These areas are a commitment to honesty and integrity, a dedication to professional obligations and commitments, collaboration, responsibility, appropriate interpersonal conduct, respect for differences, and an awareness of their own personal circumstances. The rubric defines varying levels of competence across these areas. Evaluating professionals with clearly defined observable skills and competencies can lead an organization to appoint appropriate models for the supervision of trainees. When selecting supervisors and supervisor trainers, it is important to select personnel who have scored as acceptable or exemplary across all of the soft skills for professionals. Importantly, the organization and the field would benefit from an evaluation of these soft skills that are integral to professionalism.

**Table 2:** Dispositions for professionals rubric: 1.04 integrity and 1.05 professional and scientific relationships

Dispositions rubric	Unacceptable professional practice	Emerging professional practice	Acceptable professional practice	Exemplary professional practice
Demonstrates commitment to honesty and integrity	Violates informed consent; violates confidentiality; inaccurately reports or falsifies data	Requires guidance in responsibilities regarding clients’ confidentiality and consent or requires monitoring to avoid errors in data collection, reporting, and IOA	Always obtains informed consent and protects clients’ confidentiality; consistently keeps accurate data, provides comprehensive and accurate analysis of data, and reports IOA	Supports colleagues in developing effective systems for acquiring and maintaining informed consent and confidentiality; improves record keeping, data collection, analysis, and reporting
Maintains professional obligations and commitments	Fails to follow through on contractual and professional commitments; engages in practices that lead to delays or lack of services; does not meet legal obligations	Requires some guidance in responsibilities regarding contractual and professional obligations; may need checklists or technology support guidance; requires support to consistently use practices that lead to quality work	Follows through on obligations and professional commitments and engages in practices such as timeliness with reports and assessments with high-quality work; uses the systems developed that maintain tracking and reporting	Consistently engages in actions to improve practices and provide professional development within the organization for improving tracking systems that lead to the best client outcomes
Demonstrates commitment to collaboration	Has negative or divisive relationships and interactions with colleagues and clients; addresses concerns inappropriately with disrespectful language/manner	Maintains cordial relationships with colleagues and clients using respectful language and manners when addressing areas of concern; requires problem-solving support regarding relationships within the organization, with clients, and with nonbehavioral colleagues	Has collaborative and cooperative relationships with colleagues and clients; demonstrates a positive intent and willingness to learn from others when addressing issues of concern	Initiates and facilitates collaboration with both behavioral and nonbehavioral colleagues to improve client outcomes and the professional climate in the organization
Demonstrates responsibility	Demonstrates a disregard for deadlines and systems of support to meet professional obligations; provides little to no explanation to stakeholders about implementation and plans	Needs guidance and training on organizational systems to adhere to deadlines and follow through; displays some awareness of the audience when using behavioral language	Adheres to deadlines, follows through, and communicates behavioral programs in a language that is understood by stakeholders	Takes initiative to assume responsibilities within the organization; provides professional development on individualized organizational strategies and report writing for colleagues
Demonstrates appropriate interpersonal conduct	Behaves in a manner inconsistent with the establishment of professional relationships; uses verbal communication that does not foster interaction; provides feedback in an inappropriate manner or at inappropriate times; engages in behaviors that are discriminatory or harassing	Requires some guidance on establishing relationships with colleagues and stakeholders; responds promptly to electronic communication; provides feedback appropriately with guidance; demonstrates some critical thinking and reflective skills	Has positive interactions with colleagues and stakeholders; shows evidenced-based knowledge; has established relationships; responds promptly to electronic communication; provides feedback on technical skills; demonstrates critical thinking and reflection	Has interactions with colleagues and stakeholders that encourage and facilitate interactions among members of the team, and enhance critical thinking and reflection; provides feedback on technical skills and professional dispositions
Shows respect for differences in age, gender, race, culture, ethnicity, national origin, religion, sexual orientation, disability, language, and socioeconomic status	Demonstrates no awareness of, nor concern for, bias in the development of intervention plans and engagement with clients; uses activities, language, or assessment tools that promote stereotypes; demonstrates an unwillingness to revise practice according to constructive feedback	Demonstrates a limited awareness of equitable practices, activities, and assessment tools; with support and encouragement, works to professionally develop ways to examine practice and somewhat revises practice according to constructive performance feedback	Consistently exhibits respect, ability, and awareness to select interventions, plans, and assessments that reflect cultural awareness and consideration; actively seeks training and supervision or makes appropriate referrals	Advocates for and consistently exhibits respect, ability, and awareness to select interventions, plans, and assessments that reflect cultural awareness and consideration; leads equity-based professional development; seeks outside expertise to improve equitable practices for clients and families
Shows awareness of personal circumstances	Lacks awareness of self-care and allows personal problems and conflicts to interfere with work performance and client progress; is dismissive of feedback from supervisors	Monitors issues related to self-care with prompting; understands the central role of self-care for effective practice; responds to feedback and makes changes to case load when personal problems interfere with effectiveness	Self-monitors issues related to self-care and promptly intervenes when personal problems or conflicts interfere with service delivery; takes proactive steps to protect service delivery for clients	Self-monitors issues related to self-care and promptly intervenes to prevent issues related to service delivery for clients; develops systems for the organization to monitor and support coworkers and clients with uninterrupted services

*Note.* IOA = interobserver agreement.

### Structure and Organization

When a trainee is receiving supervision through an organization, multiple individuals will likely be providing the supervision. It may benefit the organization to put in safeguards to ensure that a representative has some oversight of other assigned supervisors. Throughout the supervision of the trainee, this representative can check documentation of fieldwork experiences, including documentation of unrestricted hours, and relate these experiences back to the task list and the job model. The BACB provides the Fieldwork Tracker, which is a useful tool to document fieldwork and can be used for these purposes (BACB, 2018b). This management will ensure that the trainee and supervisor are adequately and correctly documenting experiences and that the experiences are directly related not only to the task list but also to priorities established for BCBAs working in the organization. In addition, this representative can monitor the completion of treatment integrity checklists to ensure that BST is being implemented by the supervisor and that the trainee is adequately progressing.

### Training for Supervisors

As previously noted, a BCBA may not possess all the skills needed to be an effective supervisor. An organization may invest in training these individuals to promote the reputation of the organization, improve the skills of the supervisor, provide effective supervision to the trainee, and promote positive outcomes for consumers. Best practices indicate that using a BST model is an effective method to train the trainer to competency (Parsons, Rollyson, & Reid, 2013). Other strategies that may be integrated into the training of the trainer are bug-in-the-ear (BIE) training and video modeling. BIE offers an alternative to in-person feedback. Like immediate feedback, this procedure has the benefits of fast and efficient acquisition (Scheeler, Ruhl, & McAfee, 2004) without the potential side effects of reactivity (Cooper, Heron, & Heward, 2020) or stigmatization of the client, while achieving relatively high scores on social validity measures (Artman-Meeker, Rosenberg, Badgett, Yang, & Penney, 2017). Video modeling training requires fewer resources to implement than BIE or traditional side-by-side feedback while positively affecting staff performance. Improvements have been demonstrated across multiple skills, including conducting functional analyses (Moore & Fisher, 2007) and discrete-trial training (Catania, Almeida, Liu-Constant, & DiGennaro Reed, 2009).

Once a BCBA is deemed ready to supervise, systematic follow-up on the part of the supervisor trainer may prevent behavioral drift. This can include monitoring interactions with the trainee during sessions and away from clients, as well as reviewing the supervisor’s documentation of supervision.

### Feedback to Supervisors

Sellers, Valentino, and LeBlanc (Sellers, Valentino, & LeBlanc, 2016b) recommended several methods for measuring the effects of supervision, including having trainees provide frequent feedback to their supervisors. Suggestions include soliciting both informal and formal feedback. Sellers, Valentino, and LeBlanc also recommended conducting a brief survey at the end of a supervision session that includes rating the knowledge level of the supervisor, the value of the information shared, and the level of positive statements provided. Additionally, we recommend that organizations implement a checklist for documenting the supervision meeting, including details of what is reviewed or provided (e.g., review of a client’s behavior intervention plan, supervisor’s use of BST, opportunities for trainees to ask questions). The checklist can serve as a guide to both the supervisor and the trainee as to what the supervisor should be providing and what the trainee should expect from a supervised session. Using this type of system, trainees have an opportunity to provide feedback, and the supervisor monitor has an opportunity to review the activities of the supervisor and the input from the trainee.

Figure 1 provides a guide for organizations to identify practices that will promote appropriate supervision. Ensuring that the number of supervision contacts and percentage of time for supervision of a trainee are met is an important component for meeting BACB requirements. Evaluating supervision checklists completed by both the supervisor and the trainee provide insight into the content of supervision, as well as an indication of social validity. Reviewing documentation of training, training rubrics, and treatment integrity checklists can identify the degree to which the supervisor meets organizational standards for the use of BST and confirms that all identified training areas are addressed. Providing ongoing competency checks safeguards against supervisors who do not have appropriate skills providing instruction to trainees. An organization may benefit from appointing a supervisor trainer/monitor who is responsible for all of these systems and supervisors.

## Failure to Report to the BACB

Failure to self-report or respond to the BACB as required accounted for 29 of the ethics violations and code enforcement items in the white paper (BACB, 2018a). This is found under Code Element 10.02, Timely Responding, Reporting, and Updating of Information Provided to the BACB (BACB, 2014). Self-reporting and responses to the BACB are addressed in ethics coursework and texts used in coursework, so why do these violations occur? Reasons may be that (a) it is not always clear what constitutes reportable offenses or when a response is required, (b) this issue may not be addressed in CE opportunities, (c) there are times trainees are working in the field prior to taking the ethics coursework and may not have had exposure to the BACB Code and self-reporting requirements, and (d) an employee may work in an organization that does not have a human resources department that would provide reminders regarding reporting requirements.

In order to determine what reportable violations or reporting rules are, a BCBA can read the *BACB Newsletter*, review the BACB website, and earn CE hours in ethics. However, rarely do CE courses in ethics address reporting requirements and scenarios. The *BACB Newsletter* (BACB, 2016) clarified which incidents required notification within 30 days. These were primarily incidents that involve public health- and safety-related fines or tickets, incidents that impact the competent delivery of services, incidents in which clients’ health and safety were placed at risk, and incidents that would require reporting to professional liability insurance providers, clients’ third-party payers, and/or government regulatory boards.

The April 2017 *BACB Newsletter* (BACB, 2017b) provided additional direction on reporting following Code Element 10.02. Importantly, the newsletter expanded on and provided scenarios of typical reportable matters, and scenarios that would be nonreportable. Further, the newsletter indicated that a BCBA's diagnoses, treatments, and incidents that could impact the delivery of services should be reported. The newsletter also provided a reminder to notify the BACB of email changes or address changes within 30 days. Many behavior analysts move to new positions, locations, addresses, and emails over the course of their careers. Given changes in life with jobs and addresses, it can be difficult to keep track of all of the parties requiring notification. When a BCBA renews their certification through the online system, it would benefit the BCBA, as well as the BACB, if this automatically generated a reminder of reporting requirements. The portal is set up for updating information and reporting during the renewal process. When a BCBA renews their certification, they have an opportunity to update their profile with email and address changes through the portal. However, reminders may be necessary.

Although it is the responsibility of the individual to be aware of self-reporting requirements, they may lack the resources and systems often provided by a large organization that would prompt them to follow through with these expectations. A BCBA would have been exposed to the guidelines in regard to reporting while taking their coursework in behavior analysis. The BACB website provides a visual chart for self-reporting, as well as a checklist of considerations for self-reporting (BACB, 2019b) any of the incidents or actions within 30 days. If one answers yes to even one item, they would need to self-report to the BACB ethics department. An example of an item on the checklist is related to receiving a health-and safety-related ticket that may indicate a physical or mental condition that could impact the delivery of services. For example, if an individual was convicted of driving under the influence of alcohol, that could be reportable. In addition to self-reporting, there are also considerations for reporting an alleged violation by others. Reporting steps for violations by others is discussed in detail later.

The BACB requires 4 hr of ethics CE with each renewal cycle. The content of this CE can be any content related to ethics. For comparison, in California, psychologists must have 6 hr of California law and ethics training every 2 years for renewals with required content that includes updates to the laws. A visit to the California Board of Psychology website provides important law changes (California Board of Psychology, 2020), as well as recent legislation, recent regulations, and recently approved regulations not yet in effect. It is possible that the greater specificity for CE requirements for psychologist may prepare them to respond in an ethical manner related to reporting requirements.

If the trainee is working in the field prior to completion of the ethics coursework, then the supervisor is responsible for providing the necessary information and training in regard to reporting requirements. This would come under Code Elements 5.0, Behavior Analysts as Supervisors; 5.03, Supervisory Delegation; and 10.05, Compliance with BACB Supervision and Coursework Standards (BACB, 2014).

In any organization with a human resources department, the employee would report these changes to their employer, and that could generate a reminder from the organization to report to the BACB. It would benefit the organization to have systems in place that monitor certification and renewal dates and prompt self-reporting. Organizations should be informed and aware as to whether an employee has a physical or mental condition that could put their client at risk of harm or impact the competent delivery of services. This can be captured in evaluation metrics such as the dispositions rubric presented in Table 2. Systems that survey clients and caretakers are also necessary to prevent and monitor for any incompetent and neglectful service delivery. These are more obviously reportable scenarios.

## Professionalism and Integrity

The white paper that the BACB released highlighted that 34 substantiated violations in the 2016–2017 time frame were related to professionalism and integrity (BACB, 2018a). To prevent future violations related to professionalism and integrity, BCBAs could benefit from an evaluation of demonstrated aspects of professionalism using a disposition rubric as presented in Table 2. This rubric may be used to determine areas for support or training. The specific code elements identified within the white paper include (a) 1.04, regarding integrity; (b) 1.05, related to professional and scientific relationships; (c) 7.01, regarding promoting an ethical culture; and (d) 7.02, related to ethical violations by others and risk of harm (BACB, 2018a). This section will address each of these code elements in greater detail.

### Integrity

The BACB Code defines integrity as being truthful and honest, promoting these behaviors in others, and refraining from setting up contingencies that could result in the fraudulent, illegal, or unethical behaviors of others (BACB, 2014). Given this definition, it is likely that violations related to Code Element 1.04 regarding integrity are in combination with other BACB Code violations. For example, if a BCBA has a substantiated violation related to Code Element 5.03, regarding the inappropriate delegation of activities, that would indicate that the trainee the BCBA is supporting is working outside of their competence, which would be a violation of Code Element 1.02, related to boundaries of competence. This could indicate that the supervisor is not meeting their obligations related to refraining from setting up contingencies for potentially unethical behavior on the part of the trainee. The white paper released by the BACB indicated that 145 of the 219 notices had multiple actionable violations associated with them (BACB, 2018a). These data support the hypothesis that integrity may be an area of concern in concert with other BACB Code violations.

One of the challenges with supporting professionals around the area of integrity is coming up with concrete examples of what integrity is and identifying when someone is struggling within this area. The first disposition identified within Table 2 is a commitment to honesty and integrity, which covers areas such as obtaining informed consent, maintaining confidentiality, and performing accurate data collection, analysis, and reporting.

### Professional and Scientific Relationships

Code Element 1.05, related to professional and scientific relationships, has several components embedded within it. These include (a) providing services only within the context of a defined relationship, (b) using understandable language when communicating with others, (c) ensuring culturally aware service delivery, (d) refraining from engaging in discrimination or harassment, and (e) refraining from delivering services when personal problems have the potential to interfere with the services’ effectiveness (BACB, 2014).

When BCBAs provide services within the context of a defined relationship, this means that the BCBA is refraining from delivering free advice to others (Bailey & Burch, 2016). O’Leary, Miller, Olive, and Kelly (2017) highlighted the pitfalls of breaking this code with the myriad of social media posts seeking advice from others regarding clinical issues. They provided recommendations such as stressing the fact that this is not a defined professional relationship when providing suggestions and providing recommendations regarding specific research articles that could assist with answering the questions posed. A defined professional relationship is one in which a written or verbal contract specifies the expectations around those services (Bailey & Burch, 2016). Table 2 includes a section on professional obligations and commitments that focuses on following through on contractual commitments and legal obligations.

The BACB Code highlights the importance of communicating with stakeholders by using understandable language, and Code Element 1.05 is one of those areas in which this is discussed (BACB, 2014). Table 2 provides guidance on this area through a professional’s commitment to collaboration, which includes interacting with interdisciplinary colleagues. The table also covers communication within the context of demonstrating responsibility by describing behavioral programs in an understandable way. Finally, the table addresses areas around appropriate interpersonal conduct by fostering interactions with others, responding promptly to electronic communication, and demonstrating critical thinking skills.

As mentioned previously, an area that is drawing focus for BCBAs is ensuring culturally aware service delivery, which includes understanding how our own cultural values and learning histories can impact our relationship with our clients (Fong, Catagnus, Brodhead, Quigley, & Field, 2016). Code Element 1.05 also stresses the importance of consulting another BCBA when working with a client of a different background, such as age, gender, race, culture, ethnicity, national origin, religion, sexual orientation, disability, language, or socioeconomic status (BACB, 2014). This code element also indicates that a BCBA will refrain from engaging in harassment or discrimination related to these various backgrounds. Table 2 provides guidance on evaluating a professional’s level of respect and awareness related to selecting assessments to use and making treatment recommendations with an eye toward cultural awareness. It also focuses on the degree to which this person seeks out professional development opportunities related to equity, social justice, and cultural awareness.

Finally, Code Element 1.05 focuses on refraining from providing services when personal problems are likely to compromise service delivery (BACB, 2014). It is up to the BCBAs to examine their own circumstances and proactively plan for someone else to provide services when faced with adverse life events (Bailey & Burch, 2016). Table 2 focuses on personal circumstances and the degree to which professionals focus on self-care and proactively protect service delivery for clients.

### Promoting an Ethical Culture

Code Element 7.01 states that behavior analysts have a responsibility to promote an ethical culture and to make others aware of this code (BACB, 2014). Brodhead, Quigley, and Cox (Brodhead, Quigley, & Cox, 2018b) described the value of evaluating the ethical culture of an organization prior to employment. They provided suggestions to guide applicants, including the recommendation to identify the organization’s practices related to professional development, training, and supervision; supporting an ethical culture; working within one’s scope of competency; handling health records; supporting collaboration with colleagues from different fields; and being aware of cultural differences. Organizations may need direction on how to establish and promote an ethical culture. An ethical culture starts with leadership. Ethical leaders inspire confidence and are viewed as role models by their employees (Kio, Ma, Bartnik, Haney, & Kang, 2017). Further, these employees are likely to behave ethically and to report the unethical behavior of others. By implementing practices that model and support ethical responding, the organization can create an environment in which the most common violations of the BACB Code related to supervision/delegation, reporting/responding to the BACB as required, and professionalism/integrity are avoided.

An organizational compliance monitoring checklist (Figure 1), as referenced earlier, is provided to assist organizations in achieving an ethical culture with practical recommendations for addressing Code Elements 7.01, 1.04, and 5.0. Recommendations fall into three broad categories: establishing policies, providing appropriate training, and monitoring/reinforcement.

#### Policies and Procedures

An organization’s mission statement defines the core values of the organization. Policies and procedures are then put in place to support and reflect those core values. Behavior-analytic organizations can ensure that both the mission and their policies reference the BACB Code and that procedures are carried out to support the implementation of the BACB Code. Should an incident arise where there appears to be incongruence between the BACB Code and a policy, the organization may work to understand the spirit of the BACB Code and modify its policies and procedures to eliminate the incongruence. Brodhead, Quigley, and Cox (Brodhead, Quigley, & Cox, 2018b) emphasized the importance of an organization’s values and policies aligning with the BACB Code in that this eliminates (at least initially) the opportunity for an organization to request an employee to violate the BACB Code in some way.

#### Staff Training

When organizations implement training and contingencies that support the ethical behavior of the behavior analyst, they can ensure that staff are aware of these standards, act in a way to support the best interests of clients, discriminate situations that may lead to an ethical violation, and can problem solve these situations. Brodhead and Higbee (2012) described how organizations may implement initial and ongoing training on ethics. They suggested using an ethics coordinator, developing a declaration of services (Bailey & Burch, 2016), including ethics discussions during individual supervision meetings, incorporating ethics discussions into group trainings, and developing a repository of scenarios.

Additional suggestions provided here include identifying an outside consultant whose primary source of income is not tied to the organization to serve as a trusted support to evaluate ethical concerns. In the development of ethics trainings, the organization may find it difficult to design a training program that addresses the multitude of ethical situations that an individual may encounter. However, organizations can gear ethics trainings to address common ethical concerns that arise, as well as provide an avenue for employees to bring up ethical concerns (Brodhead & Higbee, 2012). The organization may also find it difficult to bring employees together for trainings and may choose to use an asynchronous webinar format, readily available to employees on demand, with set expectations from the organization on completion of these webinars.

As discussed earlier in relation to the training of supervisors, there are other trainings that are relevant to supporting an ethical culture that organizations will want to incorporate into their professional development series. These include cultural diversity, awareness, and humility; federal and state laws; disposition; and providing information in understandable language. The most difficult of these areas may be cultural diversity, awareness, and humility. Creating an equitable culture will encompass many ongoing steps, including, but perhaps not limited to, establishing a diverse workforce, ensuring equitable practices across employees and clients, setting up an on-site task force, and bringing in experts in this area (Wright, 2020).

#### Monitoring and Reinforcement of Ethical Practices

Using a competency-based model will ensure that all BCBAs in the organization are progressing toward the organization’s goals for ethical responding. However, competent performance during training sessions is not sufficient. Although an organization may provide training, without contingencies to support ethical responding, training may not result in ethical behavior on the job. Those familiar with the research on say–do correspondence (Luciano, Herruzo, & Barnes-Holmes, 2001) will agree that simply being able to voice an ethical response does not guarantee that a BCBA will behave in an ethical manner. Daniels and Bailey (2014) highlight that training is an antecedent intervention. Although antecedent interventions may evoke a behavior, consequence strategies are necessary to maintain the behavior over time. Organizations may benefit from establishing monitoring systems for various aspects of ethical responding. Multiple examples of these tools are provided in this article.

The organization can monitor complaints (Brodhead & Higbee, 2012) and praise from clients, coworkers, and community members; request feedback from consumers on their experiences with employees; and provide observations and feedback. The organization should also recognize that there may be competing contingencies in place, and work to resolve these. Tying ethical responding into an organization’s existing reinforcement system (e.g., public recognition, entrance into a lottery, or reward) or performance-based pay plan may be an option for some organizations.

### Ethical Violations by Others and Risk of Harm

As BCBAs interact within a professional environment, they are likely to come across situations for potential legal or ethical violations. According to Code Element 7.02, BCBAs have a responsibility to determine the risk of harm and intervene accordingly (BACB, 2014). In the event that a client’s legal rights are being violated or there is a risk of harm, the BCBA should take the necessary actions to address these concerns immediately. Bailey and Burch (2016) highlighted that the BCBA should discriminate as to whether the information was observed or was communicated to them by a third party. The responses for each are different. For example, if the information is indeed firsthand and constitutes abuse or neglect, the BCBA should report this to the appropriate authorities; however, if the BCBA is told by another mandated reporter information that may constitute abuse or neglect, the BCBA should inform that individual of their obligation.

More frequently, BCBAs are likely to come across situations that can be addressed informally. In these instances, the BCBA should bring the concern to the attention of that individual and document their efforts in addressing this issue (BACB, 2014). Turner et al. (2016) identified steps for providing difficult feedback within a supervisory relationship that can be applied to this type of situation. Specifically, they recommended consulting a trusted colleague to gain support and guidance. Next, they suggested practicing the conversation ahead of time. When having the conversation, the BCBA should document it and include the date and time the conversation takes place, the event in question, the response, and the plan moving forward (Turner et al., 2016). If the BCBA determines that the issue has not been resolved, they have an obligation to report the concern to the appropriate authority, which may include reporting the issue to the BACB (BACB, 2014).

The BACB provides guidance on the decision-making process for determining if and when someone should report a potential ethics violation to them with a flowchart titled “Considerations for Reporting an Alleged Allegation” (BACB, 2019b). The flowchart indicates that a BCBA should make a report within 6 months of the time that they became aware of the concern. This time frame allows the BCBA to resolve the issue with the person directly prior to making the report. Although in some situations it is appropriate to wait, to give the BCBA ample opportunity to address the issue, other concerns may be reported sooner. For example, if there is an issue where immediate rectification is necessary to ensure the quality of services that clients receive, the BCBA may report the concern to the BACB sooner.

An organization may assist its employees in these endeavors by creating an environment where a collaborative relationship is not only accepted but also expected. The more often an individual has exposure to providing feedback with positive outcomes, the more likely that individual will continue to provide feedback. In terms of identifying a trusted colleague, ethics training at the organizational level may help an employee in discriminating peers who respond ethically from those who do not. This can also be advantageous in identifying additional supports outside one’s organization. Bailey and Burch (2016) recommended this practice, as ethical concerns often stem from practices in the organization in which the BCBA works. That is, the BCBA may need someone outside of their own organization from whom they can seek consultation and guidance. Although promoting this practice may be uncomfortable for leaders in some organizations, it can improve the ethical responding exhibited by their employees.

## Summary

Given the ever-increasing number of BCBAs joining the profession and providing services in schools, homes, and communities, it is important that behavior analysts improve their ethical training during supervision and within organizations and employment. Even with university training in ethics, ethical decision trees, access to journal articles, and evidence-based knowledge, we, as behavior analysts, know that context matters and daily practice in a structured, ethical decision-making process is important for developing fluent ethical behavior (Rosenberg & Schwartz, 2019). In this article, we have highlighted the process for teaching, practicing, and reinforcing ethical behaviors, as well as developing systems that support and maintain ethical behaviors and ethical cultures. We focused on the three most commonly identified ethical violations (BACB, 2018a): (a) improper or inadequate supervision/delegation, (b) failure to report/respond to the BACB as required, and (c) professionalism/integrity.

Supervision is required for trainees entering the field, and these practitioners may be the ones with the least amount of experience and knowledge of evidence-based methodology. They may also be the least able to assess the scope of their competence. Part of the supervision process should entail the development of self-awareness skills to determine one’s scope of competence. The supervisor’s role, in part, is as a model of ethical behavior and ethical decision making in the process of clinical problem solving. Supervisors model the skill themselves when they decide to take on a trainee and determine if they have the competence and the time given their caseload. They determine if they can serve the trainee in their specific area of focus and make the decision to supervise that particular trainee. Supervisors set up the contractual relationship, including a scope and sequence that use evidence-based methods for skill development. BST is a method of skill development with feedback that provides for the demonstration of independence prior to delegation. Evaluation of supervision and the trainee with checklists, direct measures, and social validity scales will guide and determine the effectiveness of supervision for monitoring and generalization.

Timely reporting/responding to the BACB is a common area for violations. Often these can be minor infractions that can be corrected by sending the BACB an address or email change. But these can also be more serious violations that can lead to client harm or failure to deliver competent services. We have provided potential sources of the failure to report and make some recommendations to support reporting. Importantly, the BACB provides useful visual process maps for reporting incidents and instructions for steps to be taken (BACB, 2019b).

Trainees are often employed in organizations and school districts. These entities would benefit from providing ongoing training in ethical problem solving (Brodhead & Higbee, 2012). Organizations should provide clear expectations, train to these expectations, and have written protocols in place with reinforcement for adherence to these protocols. It should be incumbent on the organization to establish and promote an ethical culture. That can be accomplished through mission statements and mission-driven services plus training and evaluations that promote adequate supervision and delegation. Ethical cultures are guided by policies and procedures that align with the BACB Code and recognize the importance of cultural diversity. Cultural awareness should be integrated into the organization’s systems with considerations for preferences and assessments, guiding service delivery, supervision, and professional development (Fong et al., 2016). Organizations need systems in place given that it is the organization that is responsible for providing supervision. Daniels and Bailey (2014) indicated that the training provided is the antecedent strategy, but often organizations can fall short on the consequent strategies—the reinforcement of professional behaviors. Organizations may need to set up clear antecedent and consequent systems. To assure more ethical responding, they may also need to monitor the technical competencies, trainings offered, and evaluation and feedback systems, as well as the integrity and professionalism of their workforce.

The BACB white paper (BACB, 2018a) provided a very clear breakdown of the most common violation categories. As we are maturing as a field, it is beneficial to continue to review, monitor, and, importantly, prevent ethical violations. This process will promote better service delivery and improve the practices of organizations and individual practitioners.
